# Supplementary material for: Macrophage VLDLR mediates obesity-induced insulin resistance with adipose tissue inflammation
Source: Nat Commun. 2017 Oct 20;8:1087. doi: 10.1038/s41467-017-01232-w (PMC5651811; doi:10.1038/s41467-017-01232-w)
Supplement: Supplementary file 1 — Supplementary Information [file 41467_2017_1232_MOESM1_ESM.pdf]

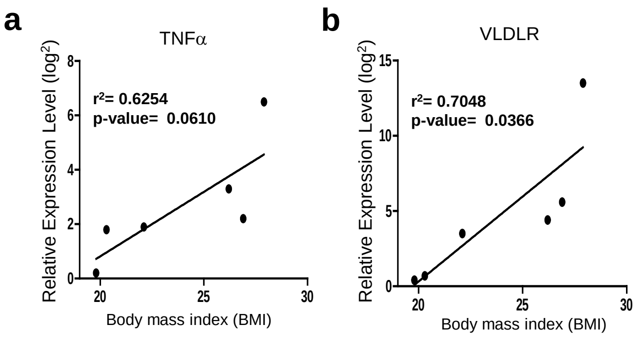

Supplementary Figure 1. The level of VLDLR mRNA shows a positive correlation with body mass index in the human fat tissue. (a, b) Correlation between mRNA levels of both TNFα (a) and VLDLR (b) genes, and body mass index (BMI) in human adipose tissue. r^2 and p-values are indicated on the graph.

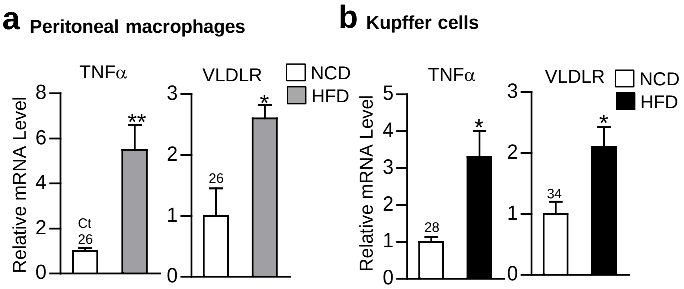

Supplementary Figure 2. The levels of VLDLR mRNA are upregulated in peritoneal and liver macrophages of obese mice. (a, b) Relative mRNA levels in peritoneal macrophages (a) and in liver macrophages (kupffer cells) (b) from NCD- or HFD-fed mice. Each mRNA level was normalized to cyclophilin mRNA. Data represent mean ± SD. $^{*}P < 0.05$, $^{**}P < 0.01$, Student's *t*-test.

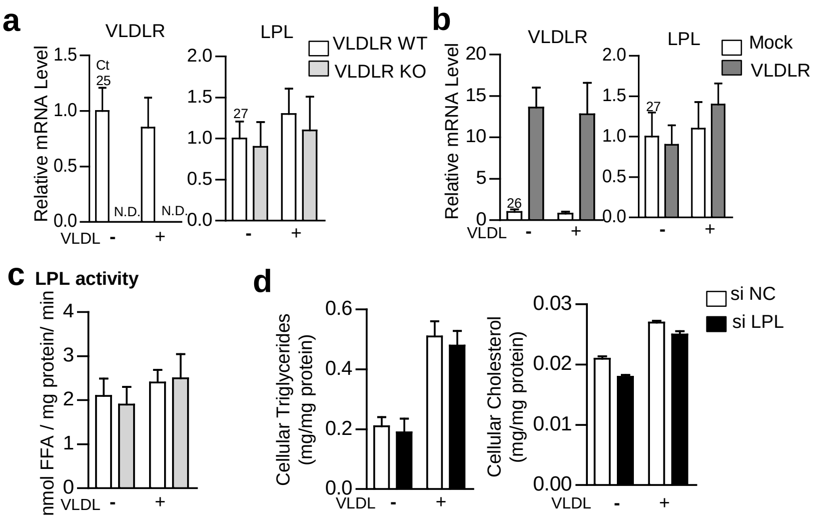

Supplementary Figure 3. Macrophage LPL is not crucial to modulate intracellular triglycerides contents with or without VLDL. (a) Relative mRNA levels of VLDLR and LPL in WT and VLDLR KO BMDMs. (b) Relative mRNA levels of VLDLR and LPL in peritoneal macrophages overexpressing VLDLR. Each mRNA level was normalized to cyclophilin mRNA. (c) LPL enzymatic activity in WT and VLDLR KO BMDMs. (d) Intracellular triglycerides and cholesterol levels in peritoneal macrophages suppressing of LPL expression with siRNA. (a-d) All the experiments were performed with or without human VLDL (30 μg/ml). Data represent mean ± SD.

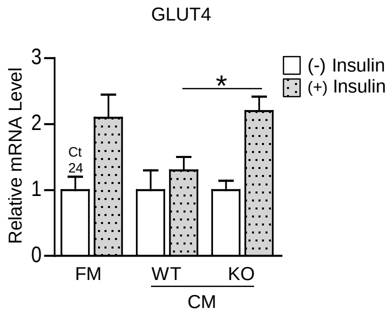

Supplementary Figure 4. The mRNA level of adipocyte GLUT4 is potentiated by CM treatment from VLDLR KO BMDMs. Relative mRNA level of GLUT4 in 3T3-L1 adipocytes. GLUT4 mRNA level was normalized to cyclophilin mRNA. Data represent mean ± SD. $^{*}P < 0.05$, Student's *t*-test.

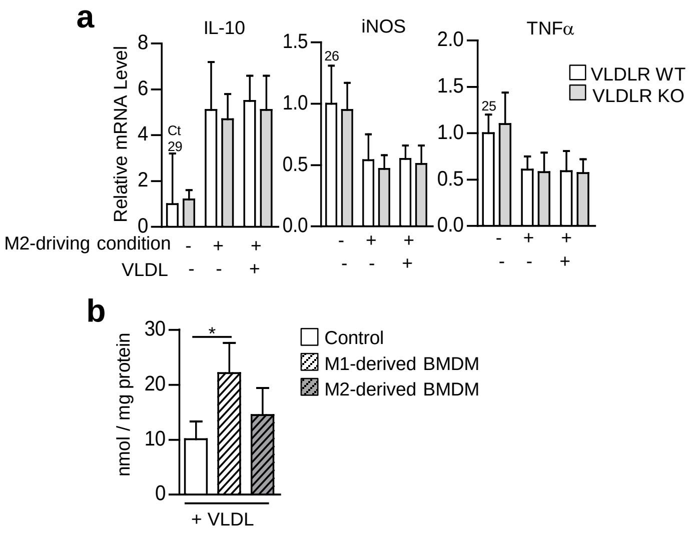

Supplementary Figure 5. Inflammatory responses are not significantly altered by VLDL nor VLDLR in the M2-derived BMDMs. (a) Relative mRNA levels of inflammatory genes in M2-derived BMDMs from WT and VLDLR KO mice with or without human VLDL (30 μg/ml). Each mRNA level was normalized to cyclophilin mRNA. (b) The levels of intracellular ceramides in M1- or M2-derived BMDMs with human VLDL (30 μg/ml). Data represent mean ± SD. $^{*}P < 0.05$ ± VLDL, Student's *t*-test.

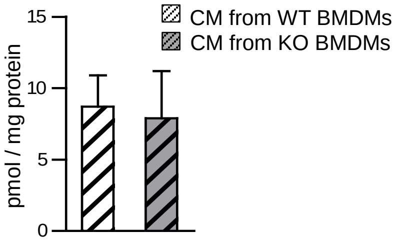

Supplementary Figure 6. The levels of secreted ceramides are not different in CM from WT and VLDLR KO macrophages. The levels of secreted ceramides in CM from WT and VLDLR KO BMDMs.

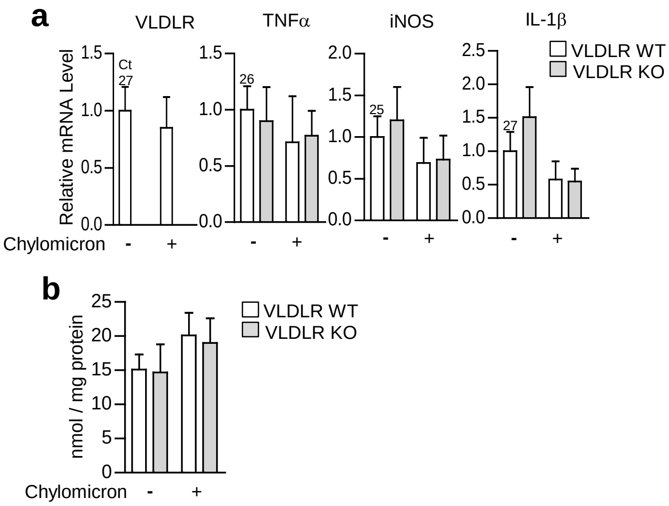

Supplementary Figure 7. Chylomicron does not influence inflammatory responses nor intracellular ceramide contents in BMDMs. (a) Relative mRNA levels of pro-inflammatory genes in WT and VLDLR KO BMDMs with or without chylomicron (30 μg/ml) treatment. Each mRNA level was normalized to cyclophilin mRNA. (b) Intracellular ceramides contents in WT and VLDLR KO BMDMs with or without chylomicron (30 μg/ml) treatment.

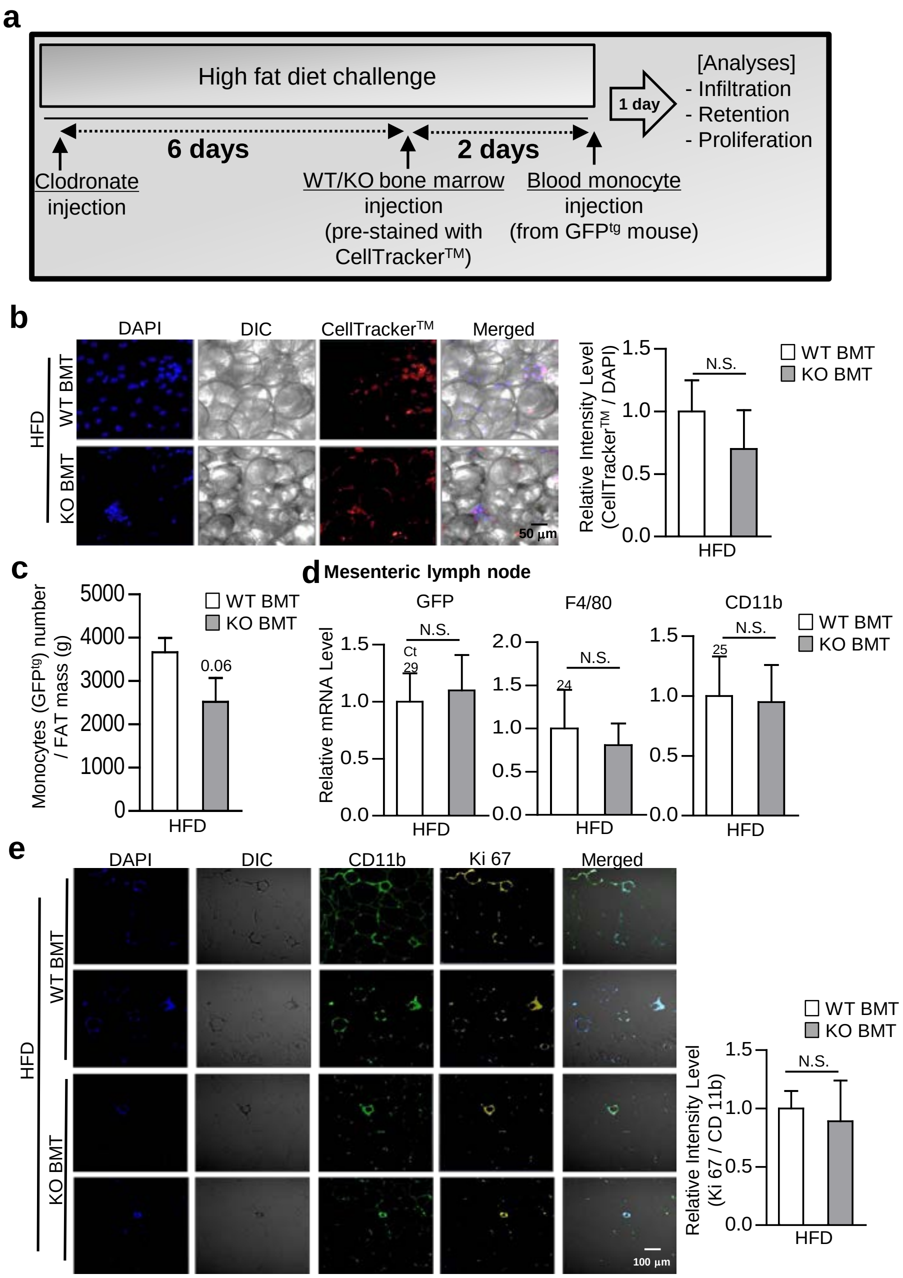

Supplementary Figure 8. Hematopoietic VLDLR deficiency attenuates macrophages accumulation in obese adipose tissue. (a) Experimental scheme. Recipient WT mice were treated with clodronate. After 6 days of clodronate treatment, isolated donor bone marrow cells from WT and VLDLR KO mice were pre-stained with CellTracker™, then adoptively transferred into recipient WT mice. Next, isolated blood monocytes from GFP transgenic mice were injected into recipient mice 2 days after transferring bone marrow cells. (b) Whole mount histochemistry analysis of nucleus (blue) and bone marrow cells (red : CellTracker™) in EATs from HFD-fed WT and KO BMT mice. Pre-stained donor bone marrow cells were adoptively transferred into WT recipient mice after 6 days of clodronate treatment. Injected donor bone marrow cells were detected in EATs of recipient mice, and the degree of transferred bone marrow cells from either WT or VLDLR KO mice was not different. (c) Infiltration of macrophages was investigated in EATs of HFD-fed WT and KO BMT mice using FACS analysis. The number of GFP^{tg} monocytes in adipose tissue mass. (d) Retention of macrophages was examined in mesenteric lymph nodes of HFD-fed WT and KO BMT mice. Relative mRNA levels of GFP gene and macrophage marker genes in mesenteric lymph nodes of HFD-fed WT and KO BMT mice. (e) The immunohistochemistry analysis of the nucleus (blue), CD11b (green), and Ki 67 (yellow), as known for cell proliferation marker gene, in EATs from HFD-fed WT and KO BMT mice. Each mRNA level was normalized to cyclophilin mRNA. Data represent mean ± SD., P = 0.06 Student's *t*-test., N.S. stands for 'not significant'.

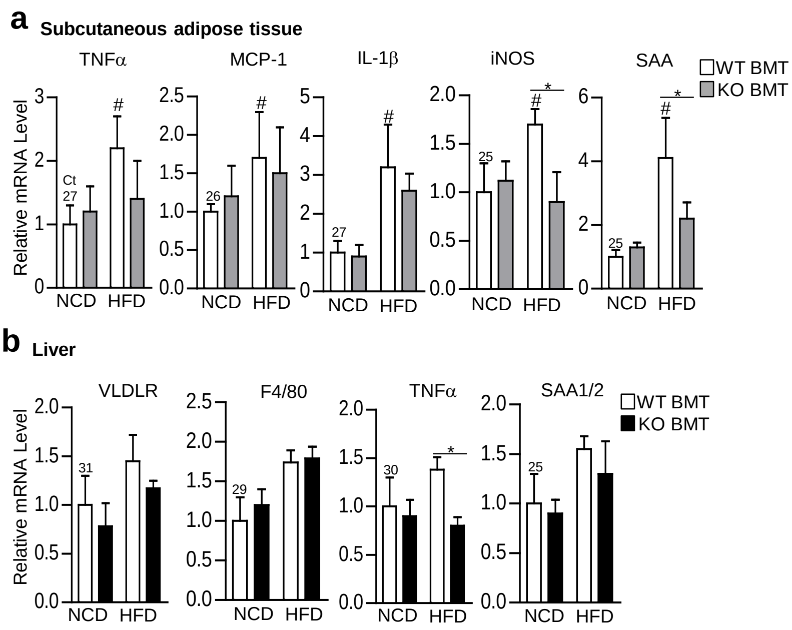

Supplementary Figure 9. Deficiency of hematopoietic VLDLR ameliorates adipose tissue inflammation in subcutaneous adipose tissues but not in liver. (a) Relative mRNA levels of pro-inflammatory genes in subcutaneous adipose tissue of NCD- or HFD-fed WT and KO BMT mice. (b) Relative mRNA levels of macrophage marker and pro-inflammatory genes in liver of NCD- or HFD-fed WT and KO BMT mice. Each mRNA level was normalized to cyclophilin mRNA. Data represent mean ± SD. $^{\#}P < 0.05$ ± HFD, $^{*}P < 0.05$, Student's *t*-test.

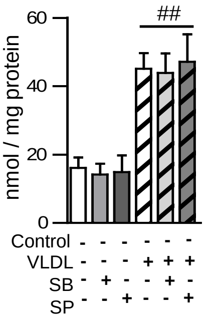

Supplementary Figure 10. Inhibition of MAPK pathways does not affect the levels of cellular ceramides in VLDL-treated BMDMs. The levels of intracellular ceramides in VLDL-treated BMDMs with or without MAPK inhibitors such as SB (SB203580, 10μM) and SP (SP600125, 10μM) treatment. Data represent mean ± SD. ##*P* < 0.01 ± VLDL, Student's *t*-test.

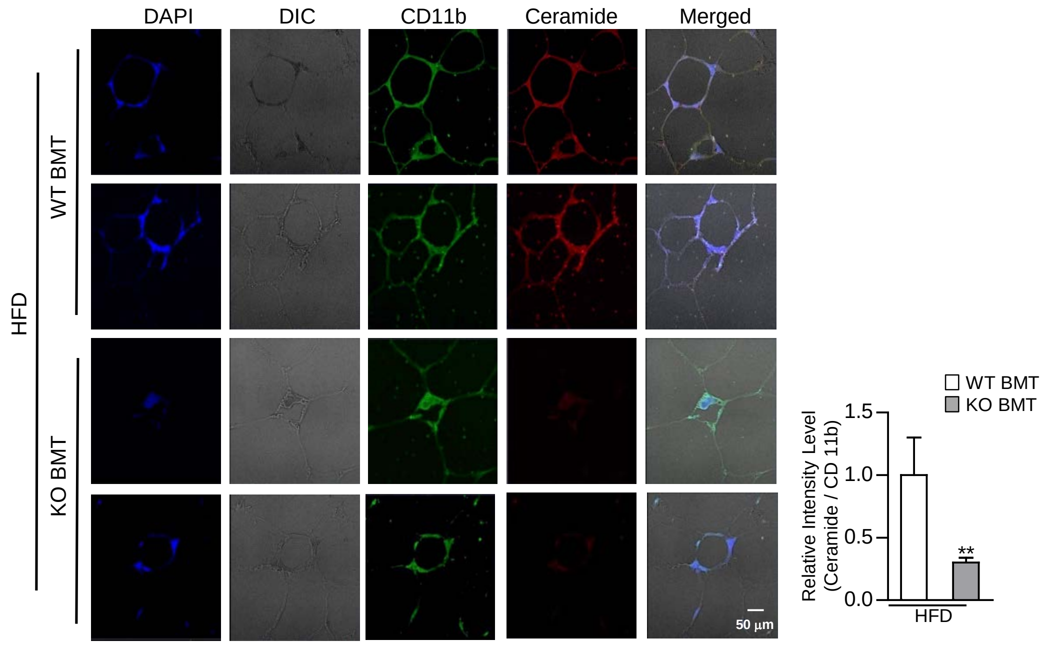

Supplementary Figure 11. The levels of ATM ceramides are reduced compared to those of HFD-fed WT BMT mice in HFD-fed KO BMT mice. The immunohistochemistry analysis of the nucleus (blue), CD11b (green), and ceramide (red) in EATs from HFD-fed WT and KO BMT mice. Data represent mean ± SD. $^{**}P < 0.01$, Student's *t*-test.

Full unedited gel for Fig. 2b

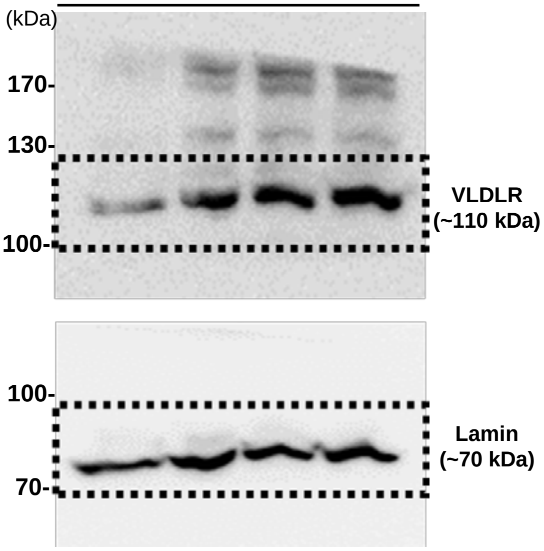

Full unedited gel for Fig. 3g

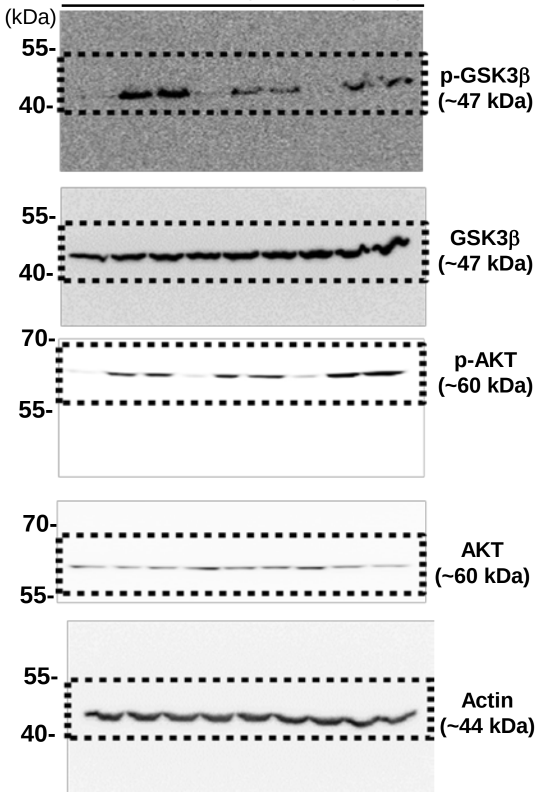

Full unedited gel for Fig. 4c

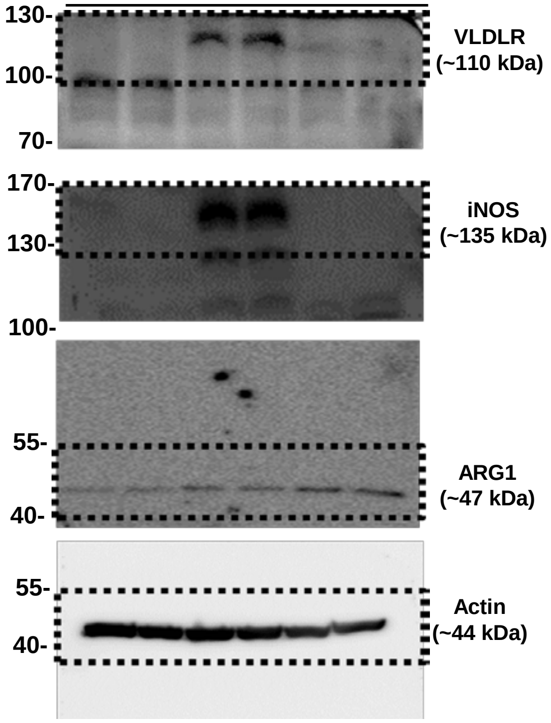

Full unedited gel for Fig. 6b

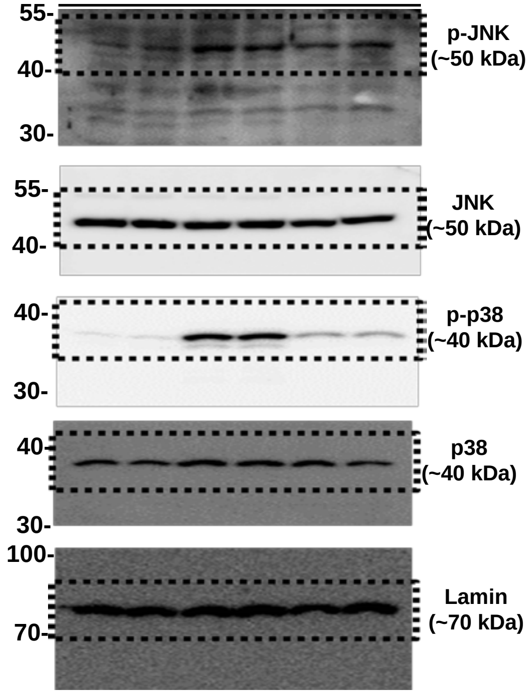

Supplementary Figure 12. The original full immunoblots presented in main figures and supplementary figures
